# The correlation of triglyceride/high-density lipoprotein cholesterol ratio with muscle mass in type 2 diabetes patients

**DOI:** 10.1186/s12902-023-01349-8

**Published:** 2023-04-26

**Authors:** Qingsong Fu, Zhenwen Zhang, Wenchao Hu, Yinrong Yang

**Affiliations:** 1 Department of Central Laboratory and Mitochondrial Medicine Laboratory, Qilu Hospital (Qingdao), Cheeloo College of Medicine, Shandong University, Qingdao, China; 2 Department of Cadre Health Care, Qilu Hospital (Qingdao), Cheeloo College of Medicine, Shandong University, Qingdao, China; 3 Department of Endocrinology, Qilu Hospital (Qingdao), Cheeloo College of Medicine, Shandong University, Qingdao, China; 4 Department of Laboratory, Qilu Hospital (Qingdao), Cheeloo College of Medicine, Shandong University, 758 Hefei Road, Shibei District, Qingdao, 266035 China

**Keywords:** Triglyceride/high-density lipoprotein cholesterol ratio, Skeletal muscle index, Type 2 diabetes mellitus

## Abstract

**Objective:**

Triglyceride/high-density lipoprotein cholesterol (TG/HDL-C) ratio is correlated with metabolic diseases. The prevalence of sarcopenia is significantly higher in type 2 diabetes mellitus (T2DM) patients compared with healthy controls. The purpose of our study is to evaluate the correlation of TG/HDL-C ratio with muscle mass in T2DM patients.

**Method:**

Our study consists of 1048 T2DM inpatients recruited from the department of endocrinology. Skeletal muscle index (SMI) was detected with a dual energy X-ray absorptiometry method. Low muscle mass was diagnosed using the criteria of SMI less than 7.0 kg/m^2^ (in male subjects) or 5.4 kg/m^2^ (in female subjects).

**Result:**

The prevalence of low muscle mass was 20.9% and 14.5% in male and female groups respectively. SMI was correlated with TG/HDL ratio after adjustment for age, duration of diabetes, diastolic blood pressure (DBP), and HbA1c in male subgroup. In female subgroup, SMI was associated with TG/HDL ratio after adjustment for age and DBP.

**Conclusion:**

Higher TG/HDL-C ratio is correlated with muscle mass in T2DM patients.

## Introduction

Sarcopenia is defined as a disease with the characteristics of progressive loss of skeletal muscle mass and strength [1]. Sarcopenia is demonstrated to increase the risk of disabilities, infection, metabolic disorders, falls and fractures, and mortality [2]. Traditional factors including aging, physical inactivity, and malnutrition are correlated with the development of sarcopenia [3]. Type 2 diabetes mellitus (T2DM), one of the most common metabolic diseases, is reported to significantly increase the risk of developing sarcopenia compared with control subjects in Korean population [4]. The prevalence of sarcopenia was dramatically higher in the T2DM patients compared with healthy controls [5].

Dyslipidemia, represented by elevated blood low-density lipoprotein cholesterol (LDL), decreased high-density lipoprotein cholesterol (HDL), and elevated blood triglyceride (TG) levels, is a well-known marker for metabolic syndrome [6], T2DM [7], and cardiovascular disease [8]. Among lipid profiles, the TG/HDL ratio has emerged as a good predictive index for insulin resistance [9], diabetes [10], and cardiovascular diseases [11]. We hypothesized that higher TG/HDL-C ratio may be correlated with muscle mass.

Therefore, the purpose of our study is to evaluate the correlation of higher TG/HDL-C ratio with muscle mass in T2DM patients.

## Materials and methods

### Patients

Our study was cross-sectional designed and enrolled 1048 T2DM inpatients recruited from the Department of Endocrinology of our hospital from September of 2017 to September of 2019. T2DM was diagnosed according to the American Diabetic Association criteria with a fasting glucose level ≥ 7.0 mmol/L or 2-hour postprandial plasma glucose level ≥ 11.1 mmol/L. Inclusion criterion was age ≥ 20 year. Patients who were pregnant, had infectious diseases, cancer, severe hip or knee osteoarthritis, and a history of stroke were excluded. This study was approved by the Hospital ethics board and all patients provided written informed consent.

### Low muscle mass definition

A dual energy X-ray absorptiometry (Hologic Discovery A, Waltham, MA, USA) was utilized to detect the skeletal muscle index (SMI). SMI was calculated with the formula of appendicular skeletal muscle mass in kg divided by the square of the body height. Low muscle mass was diagnosed using the criteria of SMI less than 7.0 kg/m^2^ (in male subjects) or 5.4 kg/m^2^ (in female subjects) [12].

### Measurements

Information of height, weight and blood pressures, the duration of diabetes, comorbidity disease history, and medications were recorded. Body mass index (BMI) was computed as weight in kilograms divided by height squared in meters (kg/m^2^). Blood was obtained from all the subjects after an overnight fasting.

### Statistical analysis

Data are displayed as means ± SD. Chi-square tests and unpaired t test were utilized to compare the statistical significance of the differences between T2DM patients with and without low muscle mass. Data were analyzed by univariate simple and multiple linear regression models looking for significant associations between SMI or TG/HDL ratio and other variables. A *P* value of less than 0.05 was considered as statistically meaningful.

## Results

### The differences between subjects with and without low muscle mass

As shown in Table 1, the prevalence of low muscle mass was 20.9% and 14.5% in male and female groups respectively. In male subjects, low muscle mass group showed higher age, HDL, HbA1c, and percentage of sulfonylureas treatment, as well as lower BMI, SMI, diastolic blood pressure (DBP), TG, and TG/HDL ratio compared with normal muscle mass group. In female subjects, age was increased, whereas BMI, SMI, TG, TG/HDL ratio, and percentage of metformin treatment were decreased in low muscle mass group compared with normal muscle mass group.

**Table 1:** The characteristic differences between T2DM patients with and without low muscle mass

Characteristics	Male (n = 558)			Female (n = 490)		
	Normal muscle mass (n = 441)	Low muscle mass (n = 117)	*P* value	Normal muscle mass (n = 419)	Low muscle mass (n = 71)	*P* value
Age (years)	54.73 ± 12.07	60.78 ± 13.53	< 0.001	61.13 ± 10.97	65.52 ± 11.35	0.002
Duration (years)	8.04 ± 6.82	8.93 ± 5.79	0.192	8.65 ± 5.97	9.24 ± 6.75	0.446
BMI (kg/m^2^)	27.4 ± 4.74	23.58 ± 5.43	< 0.001	27.14 ± 5.03	22.17 ± 4.1	< 0.001
SMI	8.19 ± 1.54	6.45 ± 0.48	< 0.001	6.5 ± 0.89	5.07 ± 0.33	< 0.001
SBP (mmHg)	140.83 ± 19.96	138.13 ± 20.75	0.197	143.23 ± 20.87	143.07 ± 22.11	0.953
DBP (mmHg)	82.61 ± 13	78.97 ± 12.85	0.007	76.54 ± 12.87	76.45 ± 11.16	0.958
TG (mmol/L)	1.83 ± 1.08	1.39 ± 0.77	< 0.001	1.72 ± 0.94	1.39 ± 0.69	0.006
TC (mmol/L)	4.44 ± 1.04	4.45 ± 1.08	0.912	4.65 ± 1.16	4.6 ± 1.17	0.753
LDL (mmol/L)	2.97 ± 0.9	2.97 ± 0.95	0.962	3.01 ± 0.92	2.89 ± 0.99	0.319
HDL (mmol/L)	1.14 ± 0.26	1.22 ± 0.3	0.005	1.28 ± 0.32	1.36 ± 0.35	0.051
FPG (mmol/L)	8.02 ± 2.65	7.94 ± 3.17	0.766	7.74 ± 2.9	7.04 ± 2.76	0.075
HbA1c (%)	8.41 ± 2.1	8.86 ± 2.09	0.043	8.29 ± 2.02	8.19 ± 2.13	0.697
TG/HDL ratio	1.74 ± 1.15	1.26 ± 0.87	< 0.001	1.49 ± 0.16	1.12 ± 0.7	0.011
Cardiovascular disease (n, %)	105 (23.8%)	34 (29.1%)	0.243	156 (37.2%)	30 (42.3%)	0.42
Renal disease (n, %)	9 (2%)	2 (1.7%)	0.819	15 (3.6%)	3 (4.2%)	0.789
Pulmonary disease (n, %)	32 (7.3%)	9 (7.7%)	0.872	13 (3.1%)	4 (5.6%)	0.281
Treatment						
Metformin (n, %)	302 (68.5%)	75 (64.1%)	0.335	302 (72.1%)	42 (59.2%)	0.022
Acarbose (n, %)	150 (34%)	41 (35%)	0.86	166 (39.6%)	24 (33.8%)	0.337
Sulfonylureas (n, %)	105 (23.8%)	44 (37.6%)	0.003	127 (30.3%)	21 (29.6%)	0.872
DPP-IV inhibitor (n, %)	87 (19.7%)	32 (27.4%)	0.078	75 (17.9%)	12 (16.9%)	0.825
Insulin (n, %)	167 (37.9%)	44 (37.6%)	0.959	162 (38.7%)	20 (33.8%)	0.435
Statin (n, %)	123 (27.9%)	37 (31.6%)	0.603	138 (32.9%)	28 (39.4%)	0.597

### The association between SMI and other characteristics

As shown in Table 2, SMI was correlated with age, duration of diabetes, DBP, TG, HDL, HbA1c, and TG/HDL ratio in male subjects after simple linear regression analysis. Age, DBP, HbA1c, and TG/HDL ratio were still correlated with SMI after a multiple linear regression analysis.

**Table 2:** The association between clinical characteristics and SMI in male subjects

	simple regression analysis		multiple regression analysis	
	β (95% CI)	*P* value	β (95% CI)	*P* value
Age (years)	-0.27 (-0.037, -0.017)	< 0.001	-0.023 (-0.035, -0.012)	< 0.001
Duration (years)	-0.02 (-0.04, -0.001)	0.04	-0.007 (-0.027, 0.014)	0.529
SBP (mmHg)	0.005 (-0.002, 0.011)	0.137		
DBP (mmHg)	0.017 (0.007, 0.027)	0.001	0.01 (0.000, 0.021)	0.049
TG (mmol/L)	0.29 (0.167, 0.413)	<0.001	-	-
TC (mmol/L)	0.028 (-0.096, 0.151)	0.659		
LDL (mmol/L)	0.033 (-0.109, 0.176)	0.645		
HDL (mmol/L)	-0.561 (-1.035, -0.088)	0.02	-	-
FPG (mmol/L)	0.013 (-0.034, 0.059)	0.596		
HbA1c (%)	-0.071 (-0.136, -0.006)	0.032	-0.091 (-0.155, -0.028)	0.005
TG/HDL ratio	0.262 (0.148, 0.377)	< 0.001	0.177 (0.054, 0.3)	0.005

In female subjects, SMI was correlated with age, DBP, and TG/HDL ratio after simple linear regression analysis (Table 3). Multiple linear regression analysis showed that age, DBP, and TG/HDL ratio were still correlated with SMI (Table 3).

**Table 3:** The association between clinical characteristics and SMI in female subjects

	simple regression analysis		multiple regression analysis	
	β (95% CI)	*P* value	β (95% CI)	*P* value
Age (years)	-0.019 (-0.027, -0.012)	< 0.001	-0.017 (-0.025, -0.01)	< 0.001
Duration (years)	0.001 (-0.014, 0.014)	0.974		
SBP (mmHg)	0.003 (-0.001, 0.007)	0.191		
DBP (mmHg)	0.011 (0.004, 0.017)	0.002	0.007 (0.000, 0.014)	0.037
TG (mmol/L)	-0.005 (-0.079, 0.07)	0.903	-	-
TC (mmol/L)	-0.006 (-0.07, 0.058)	0.857		
LDL (mmol/L)	0.011 (-0.082, 0.103)	0.822		
HDL (mmol/L)	-0.386 (-0.651, -0.121)	0.004	-	-
FPG (mmol/L)	0.006 (-0.025, 0.037)	0.691		
HbA1c (%)	-0.009 (-0.053, 0.035)	0.691		
TG/HDL ratio	0.103 (0.026, 0.018)	0.009	0.084 (0.009, 0.159)	0.028

### The association between TG/HDL ratio and other characteristics

Simple linear regression analysis showed that TG/HDL ratio was correlated with age, duration of diabetes, BMI, DBP, total cholesterol, LDL, and fasting plasma glucose (FPG) in male subjects (Table 4). Age, duration of diabetes, BMI, and FPG were still correlated with TG/HDL ratio after a multiple linear regression analysis (Table 4).

**Table 4:** The association between TG/HDL ratio and other clinical characteristics in male subjects

	simple regression analysis		multiple regression analysis	
	β (95% CI)	*P* value	β (95% CI)	*P* value
Age (years)	-0.022 (-0.029, -0.015)	<0.001	-0.014 (-0.022, -0.007)	< 0.001
Duration (years)	-0.026 (-0.04, -0.012)	<0.001	-0.018 (-0.032, -0.004)	0.009
BMI (kg/m^2^)	0.063 (0.046, 0.081)	<0.001	0.062 (0.044, 0.08)	< 0.001
SBP (mmHg)	0.004 (-0.001, 0.009)	0.084		
DBP (mmHg)	0.011 (0.004, 0.018)	0.002	0.000 (-0.007, 0.007)	0.91
TC (mmol/L)	0.134 (0.046, 0.222)	0.003	0.166 (-0.035, 0.368)	0.106
LDL (mmol/L)	0.137 (0.036, 0.239)	0.008	-0.167 (-0.4, 0.066)	0.16
FPG (mmol/L)	0.065 (0.032, 0.098)	<0.001	0.049 (0.016, 0.081)	0.003
HbA1c (%)	0.031 (-0.015, 0.076)	0.186		

As shown in Table 5, TG/HDL ratio was correlated with FPG and HbA1c in female subjects after simple linear regression analysis. Multiple linear regression analysis showed that FPG was still correlated with TG/HDL ratio.

**Table 5:** The association between TG/HDL and other clinical characteristics in female subjects

	simple regression analysis		multiple regression analysis	
	β (95% CI)	*P* value	β (95% CI)	*P* value
Age (years)	-0.007 (-0.016, 0.002)	0.123		
Duration (years)	0.008 (-0.008, 0.024)	0.343		
BMI (kg/m^2^)	0.014 (-0.005, 0.034)	0.151		
SBP (mmHg)	0.001 (-0.003, 0.006)	0.581		
DBP (mmHg)	0.007 (0.000, 0.015)	0.066		
TC (mmol/L)	-0.013 (-0.099, 0.073)	0.766		
LDL (mmol/L)	0.027 (-0.079, 0.134)	0.617		
FPG (mmol/L)	0.088 (0.053, 0.122)	<0.001	0.072 (0.029, 0.115)	0.001
HbA1c (%)	0.087 (0.037, 0.137)	0.001	0.032 (-0.027, 0.092)	0.281

## Discussion

Our investigation indicated higher TG/HDL-C ratio is negatively correlated with muscle mass in T2DM patients. Wang also demonstrated that TG/HDL-C ratio was negatively associated with sarcopenia occurrence rate in community-dwelling Chinese adults [13]. However, other studies performed in Korea and Japan reported inconsistent results. The prevalence of low muscle mass significantly increased in accordance with TG/HDL ratio quartiles in elderly Korean males [14]. The atherogenic dyslipidemia ratio [log(TG)/HDL-C] was significantly related to skeletal sarcopenia in T2DM females of Japan [15]. The reason for these conflicting data is unclear but may be due to differences in disease advancement, ethnic populations or assays applied.

Our results indicated that HDL was significantly higher in low muscle mass group of T2DM patients than in normal muscle mass group. HDL was negatively correlated with SMI in T2DM patients. Tuzun reported that muscle-related index was inversely associated with HDL [16]. A cross-sectional study performed in Brazil demonstrated that HDL was higher in T2DM patients with sarcopenia compared with non-sarcopenia group [17]. In addition, other investigators also reported higher HDL in that sarcopenia group showed than in non-sarcopenia group among T2DM patients; however, the difference was not statistically meaningful [18–20]. Previous investigations showed that T2DM patients with sarcopenia had lower TG levels than those without sarcopenia [17–20]. Our investigation arrived at similar conclusions. This may be contradicted with the traditional beliefs. Obesity and hyperlipidemia are considered to be risk factors of developing metabolic disease such as diabetic complication and cardiovascular disorders. But when it comes to sarcopenia or low muscle mass, obesity and hyperlipidemia are associated with a lower risk of sarcopenia or low muscle mass development in T2DM patients. In addition, TG/HDL-C ratio may be utilized to be a biomarker to diagnose or predict low muscle mass in T2DM patients.

The present study had several limitations. First of all, the sample size of this study was relatively small. Secondly, this study was a cross-sectional study, which limited its causal conclusions. Causality must be assessed by further longitudinal researches.

## Conclusions

In conclusion, higher TG/HDL-C ratio is negatively correlated with low muscle mass in type 2 diabetes patients.

## Data Availability

The datasets used during the current study available from the corresponding author on reasonable request.
